# Evaluation of a Revised Point-of-Care Test for the Detection of Feline Leukaemia p27 Antigen and Anti-p15E Antibodies in Cats

**DOI:** 10.3390/v16040614

**Published:** 2024-04-15

**Authors:** Juliana Giselbrecht, Stéphanie Jähne, Michèle Bergmann, Marina L. Meli, Svenja Teichmann-Knorrn, Yury Zablotski, Maria-Grazia Pennisi, Nicolas Layachi, Rodrigo Serra, Stefano Bo, Regina Hofmann-Lehmann, Katrin Hartmann

**Affiliations:** 1 LMU Small Animal Clinic, Centre for Clinical Veterinary Medicine, 80539 Munich, Germany; 2 Clinical Laboratory, Department of Clinical Diagnostics and Services, and Center for Clinical Studies, Vetsuisse Faculty, University of Zurich, 8057 Zurich, Switzerland; 3 Veterinary Clinic Oberhaching, 82041 Oberhaching, Germany; 4 Department of Veterinary Sciences, University of Messina, 98168 Messina, Italy; 5 Layachi Veterinary Clinic, 33300 Bordeaux, France; 6 Investigacao Veterinaria Independente, 1700-119 Lisbon, Portugal; 7 Ambulatorio Veterinario Bo-Ferro, 10123 Turin, Italy

**Keywords:** feline leukaemia virus, FeLV, PoC test, test performance, v-RetroFel^®^, progressive infection, regressive infection, abortive infection, focal infection, antibody detection, in-house testing

## Abstract

The first point-of-care (PoC) test (v-RetroFel^®^; modified version 2021) determining the presence of FeLV p27 antigen and FeLV anti-p15E antibodies has become recently commercially available to identify different feline leukaemia virus (FeLV) infection outcomes. This study aimed to assess this PoC test’s performance concerning FeLV p27 antigen and FeLV anti-p15E antibody detection. Sensitivity, specificity, positive and negative predictive values (PPV, NPV) were assessed after ten minutes (recommended) and 20 min (prolonged) incubation times. The test results were evaluated as either positive or negative. Serum samples from 934 cats were included, originating from Italy (n = 269), Portugal (n = 240), Germany (n = 318), and France (n = 107). FeLV p27 antigen and anti-p15E antibodies were measured by reference standard ELISAs and compared to the PoC test results. The PoC test was easy to perform and the results easy to interpret. Sensitivity and specificity for FeLV p27 antigen were 82.8% (PPV: 57.8%) and 96.0% (NPV: 98.8%) after both, ten and 20 minues of incubation time. Sensitivity and specificity for anti-p15E antibodies were 31.4% (PPV: 71.6%) and 96.9% (NPV: 85.1%) after ten minutes incubation time; sensitivity was improved by a prolonged incubation time (20 min) to 40.0% (PPV: 76.3%), while specificity remained the same (96.9%, NPV: 86.7%). Despite the improved sensitivity using the prolonged incubation time, lower than ideal sensitivities for both p27 antigen and especially anti-p15E antibodies were found, indicating that the PoC test in its current version needs further improvement prior to application in the field.

## 1. Introduction

Feline leukaemia virus (FeLV) is a gammaretrovirus with worldwide distribution and is regarded as one of the most important infectious agents in cats. FeLV can cause different courses of infection, including progressive, regressive, abortive, and focal (atypical) infections. Detection of these infection outcomes can be challenging and several tests are necessary [1,2,3].

Over the past few decades, FeLV prevalence of progressively infected cats has declined in many countries due to vaccination programs and improved veterinary care, including better diagnostic techniques and testing and separating programs [4,5,6,7,8]. For example, in Germany, a constant decrease in the FeLV infection rate from 6% to 1% was observed over a period of ten years [7]. However, more recent studies suggest that the decline in prevalence has now plateaued in many countries, potentially due to incomplete vaccination coverage, evolution of the virus itself, and persistence in high-risk groups [8,9,10]. Therefore, it is crucial not to neglect awareness about this important feline infection and its prevention.

In progressive FeLV infection, the immune system of affected cats is unable to control virus replication, and this results in persistent viraemia. During the viraemic phases, free p27 antigen can be readily detected by commercially available point-of-care (PoC) tests that are based on enzyme-linked immunosorbent (ELISA) or immunomigration principles. These PoC tests detect progressive infection as well as some cats with focal and early regressive infection, but other courses of infection without viraemia (abortive and regressive infection without viraemia) remain undetected. However, abortive and regressive infections also play an important epidemiological role. Knowledge of the presence of a regressive infection is clinically relevant since this form of infection can progress to a progressive outcome with host immunosuppression. This is one reason why a reliable PoC test would be useful for rapid identification of regressively infected cats [1,2,3]. Furthermore, the easy identification of regressive infection is of particular importance in potential blood donor cats, as they can transmit FeLV provirus to FeLV-naive cats via blood transfusion [11]. Moreover, the detection of cats previously exposed to FeLV (abortive infection) prior to vaccination might be helpful in the decision whether to vaccinate cats against FeLV or not.

The FeLV transmembrane protein p15E is located on the surface of infected cells and enables the virus to enter the host cell. Additionally, it possesses immunosuppressive properties that can inhibit lymphocyte proliferation and T-cell functions [12,13]. Anti-p15E antibodies have limited virus-neutralizing properties. Lutz and colleagues (1980) found that cats that became immune or viraemic after FeLV infection showed elevated levels of anti-p15E antibodies [12,14,15]. Regardless of whether cats develop immunity (regressive and/or abortive courses) or remain viraemic following infection (progressive course), they consistently had elevated levels of antibodies against p15E [14,15,16]. P15E antibody testing can therefore be useful to identify different courses of FeLV infection. A PoC test for the detection of anti-15E antibodies (and FeLV p27 antigen) has been commercially available since 2018. The performance of this PoC test (in its original version) was evaluated with samples from 370 naturally infected cats in Australia and Germany [17]. The study demonstrated the test’s ability to accurately determine the correct FeLV infection status in 271 out of 370 cases (73.2%). However, it was not able to correctly identify most of the regressive and abortive infections. With samples from Australia, the sensitivity and specificity for anti-p15E antibodies were 16.7% and 90.2%, with samples from Germany, only 8.3% and 93.7%, respectively. Most of the progressively infected cats (92.6%; 25/27) were identified correctly by detection of p27 antigen. However, none of the samples from Australia and only one of the samples from Germany (from regressively and abortively infected cats that tested positive in a laboratory-based anti-p15E ELISA) were identified as true-positive by the PoC test. Consequently, the authors advised that use of this PoC test could not be recommended until improvements are made to increase the sensitivity [17].

The aim of the present study was to evaluate the practicability and performance of a modified new version of the commercially available PoC test (version 2021) detecting FeLV p27 antigen and anti-p15E antibodies. The evaluation of such a test prior to application in the field is of importance since clinical decisions including identification of regressively infected cats, consideration of the need for FeLV vaccination, or the suitability of cats as blood donors could rely on these test results.

## 2. Materials and Methods

### 2.1. Samples

#### 2.1.1. Experimentally Infected Cats

In total, 30 uninfected specific pathogen-free (SPF) cats and 30 experimentally FeLV-infected SPF cats (16 progressively and 14 regressively infected cats) were included. For each cat, p27 antigen, proviral deoxyribonucleic acid (DNA), and anti-p15E-antibodies in blood were known. All SPF cats included in this study had been part of experimental studies officially approved by the veterinary office of the Swiss Canton of Zurich (11/2011, 160/2010 and 251/2013).

#### 2.1.2. Naturally Infected Cats

In total, 934 cats from four different European countries (Italy n = 269, Portugal n = 240, Germany n = 318, France n = 107) were included in this study. Blood samples were collected from cats that were presented to veterinary clinics for various reasons from 2019 to 2021. The cats needed to have a minimum body weight of 1 kilogram (kg) (inclusion criterium). A subset of the blood samples has been used in a previous epidemiological study [18]. Vaccination status was determined by a survey of the owners and/or veterinarians. The present study was approved by the ethical committee of the Centre for Clinical Veterinary Medicine of the LMU Munich, Germany (reference number 142-25-08-2018).

### 2.2. Laboratory Tests

From each cat, serum and ethylenediaminetetraacetic acid (EDTA) anticoagulated blood samples were stored at −80 °C for a maximum of 24 months before being sent on dry ice to the Clinical Laboratory at the Vetsuisse Faculty, University of Zurich, where samples were tested for p27 antigen, proviral DNA, and anti-p15E antibodies.

#### 2.2.1. Detection of Free FeLV p27 Antigen in Serum

The presence of free p27 antigen in serum samples was evaluated using a sandwich ELISA as described previously [19]. Each sample was examined in duplicate, and a microplate reader (Synergy H1, Biotek, Winooski, VT, USA) was used to read the absorbances. Any value higher than 4% of the positive control (serum samples from cats naturally infected with FeLV) was classified as positive [20]. The ELISA and the monoclonal antibodies used in this assay have been evaluated and described extensively [21,22,23,24]. The ELISA is able to detect between 100 ng and >2000 ng of FeLV p27 per ml of serum in viraemic cats; the lower limit of detection corresponds to an absolute amount in a sample of 1 ng [21]. The ELISA is highly specific due to the monoclonal nature of the three antibodies; they recognized p27 in several hundred FeLV isolates but did not react with any of eight purified leukaemia viruses other than FeLV [22,23,24].

#### 2.2.2. Detection of FeLV Proviral DNA in Blood

FeLV proviral DNA was determined in all samples by extracting total nucleic acids (TNA) [25] from 100 µL of EDTA anticoagulated whole blood using the MagNA Pure 96 instrument (Roche Diagnostics AG, Rotkreuz, Switzerland) and the Viral NA SV Kit (Roche Diagnostics AG, Rotkreuz, Switzerland), following the protocol as described before [17]. The proviral DNA copy number was quantified by real-time quantitative polymerase chain reaction (qPCR) as described previously [26]. To verify the quality and quantity of the TNA a qPCR was performed for the detection of feline albumin on all 934 TNA samples as previously described [27]. In a previous study, it was shown that FeLV provirus quantitative real-time PCR showed a high analytical sensitivity (detection of 1 copy/PCR) and a high analytical specificity (detection of all three FeLV subtypes, no false-positive results in SPF cats) [28].

#### 2.2.3. Detection of FeLV Anti-p15E Antibodies in Serum

An in-house developed ELISA was used for the detection of anti-p15E antibodies in serum samples [25]. Each assay included positive and negative controls. Negative controls consisted of sera from SPF cats, while positive controls consisted of pooled serum samples from cats experimentally infected with FeLV-A/Glasgow-1. The relative optical density (ROD) values were determined using the formula ROD = [(sample optical density (OD) − negative control OD)/(positive control OD − negative control OD)]. Samples from experimentally and naturally FeLV-infected cats with ROD values >4.9% and >16.3%, respectively, compared to the positive control [25], were considered anti-p15E antibody-positive. In experimentally infected cats, the p15E ELISA showed a diagnostic sensitivity of 95.7% and a specificity of 100.0%. In naturally infected cats, the p15E ELISA showed a diagnostic sensitivity of 77.1% and a specificity of 85.6% when compared to provirus PCR results [25].

### 2.3. Classification of Courses of Infection and Vaccination Status

The classification of the 934 cats into the different courses of infection is shown in Table 1.

### 2.4. Point-of-Care Test (v-RetroFel^®^)

The PoC test, an immunochromatographic assay, was coated with antibodies specific to FeLV p27 antigen as well as with an antigen specific for FeLV p15E antibodies. In addition, the test also provided results for FIV (feline immunodeficiency virus) antibodies; evaluation of the detection of FIV antibodies was not part of this study. The PoC test was stored between 18–22 °C and carried out with serum samples according to the manufacturer’s instructions. All samples were tested with the same batch of the PoC test (ID 25110011221). Each test was incubated and interpreted according to the manufacturer’s specification after ten minutes. Additionally, each test was interpreted after a prolonged incubation period of 20 min. All samples were tested for FeLV p27 antigen and anti-p15E antibodies using the PoC test by the same investigator (J.G.) who was blinded to the results of the p27 antigen and anti-p15E antibodies ELISAs. If the result was uncertain, a second independent person assessed the results (the two investigators always agreed). Once a line was visible, regardless of the intensity of the colour, the test was considered positive.

Practicability, difficulties in test result interpretation, sensitivity (true positive rate), specificity (true negative rate), negative predictive value (NPV) (proportion of predicted negatives that were true negatives), and positive predictive value (PPV) (proportion of predicted positives that were true positives) were calculated.

### 2.5. Statistical Analysis

Laboratory data from all cats were analysed using Excel (Microsoft Germany GmbH, München, Germany) and R statistical language (version 4.1.2; R Core Team, 2020). To assess the normality of metric variables for the statistical analysis, Shapiro–Wilk normality test was conducted. McNemar test was used to compare the results of the point-of-care test after ten and 20 min of incubation time, concerning anti-p15E antibodies. Kruskal–Wallis test (“ggstatsplot” R package) was used to determine any statistically significant differences between the means of anti-p15E antibody levels in true positive, true negative, false positive and false negative samples in PoC test [30]. Holm–Bonferroni was used for the adjustment of the *p*-values to correct for multiple comparisons. For all analyses, 95% confidence intervals (CI) were calculated. A *p*-value < 0.05 was used to determine statistical significance.

## 3. Results

### 3.1. Performance of the PoC Test

**Figure 1:**
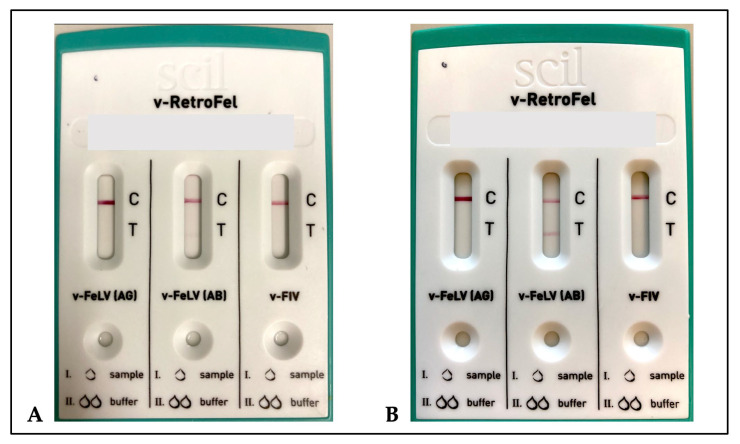
Pictures of a point-of-care test (v-RetroFel^®^, scil animal care company GmbH, Viernheim, Germany) for the detection of feline leukaemia virus p27 antigen (v-FeLV (AG)), feline leukaemia virus anti-p15E antibodies (v-FeLV (AB)), and feline immunodeficiency virus antibodies (v-FIV). The point-of-care tests were carried out with serum samples with an incubation time of ten minutes each. The pictures show a point-of care tests with a faintly coloured (picture (**A**)) and a clearly visible (picture (**B**)) test line (T) in an enzyme-linked immunosorbent assay anti-p15E antibody-positive sample (v-FeLV (AB)) as well as clearly visible control lines (C).

The PoC test can be stored at room temperature and was therefore immediately ready for use. It was easy to perform, without the need for advanced laboratory equipment or trained personnel. After ten minutes of incubation time, 36.2% (21/58) of the samples positive in the p15E antibody PoC test strips showed rather faintly coloured test lines (Figure 1), barely visible and thus difficult to interpret. When the incubation time was prolonged to 20 min, 13.8% (8/58) of the samples positive in the PoC test showed rather faintly coloured lines. In contrast, the p27 antigen test strip was easy to interpret for all samples after both ten and 20 min.

### 3.2. FeLV p27 Antigen

Among the 60 SPF cats (30 uninfected and 30 experimentally FeLV-infected), the 16 cat with progressive infection (26.7%) were p27 antigen positive. The prevalence of p27 antigen (determined by reference standard ELISA) was 6.2% (58/934) in naturally infected cats, 10.0% (27/269) in Italy, 5.4% (13/240) in Portugal, 4.7% (15/318) in Germany, and 2.8% (3/107) in France. As per definition, the prevalence of antigenemia was 100.0% (38/38) in progressively infected cats and 100.0% (11/11) in focally infected cats. The prevalence in regressively, abortively, and FeLV-unexposed cats was 0.0%. The p27 antigen performance parameters of the PoC test in experimentally infected cats and in naturally infected cats are summarized in Table 2 and Table 3. The p27 antigen performance parameters of the PoC test considering different courses of infection are given in Table 4. All test results remained identical after 20 min of incubation time. Thus, all performance parameters remained unchanged compared to ten minutes of incubation time.

### 3.3. Anti-p15E Antibodies

The prevalence of anti-p15E antibodies in the reference ELISA, when considering a ROD of >4.9% as positive for experimentally infected cats and >16.3% as positive for naturally infected cats, was 100.0% in experimentally infected cats and 19.8% (185/934) in naturally infected cats, 21.1% (57/269) in Italy, 22.5% (54/240) in Portugal, 14.1% (45/318) in Germany, and 27.1% (29/107) in France. All SPF cats (30/30) tested negative in the anti p15E antibody ELISA as well as negative in the PoC test. The anti-p15E antibody performance parameters of the PoC test in experimentally and naturally infected cats are summarized in Table 5 and Table 6. The prevalence of anti-p15E antibodies was 78.9% (30/38) in progressively infected cats, 65.0% (26/40) in regressively infected, 100.0% (108/108) in abortively infected, and 23.8% (5/21) in focally infected. Cats unexposed to and not vaccinated against FeLV showed no anti-p15E antibodies (0.0%; 0/655). In total, 22.2% (16/72) of FeLV-unexposed cats but previously vaccinated against FeLV showed anti-p15E antibodies. Four of the vaccinated cats that tested positive in the p15E ELISA also tested positive in the PoC test (25.0%; 4/16). A summary of the performance parameters of the PoC test in progressively, regressively, abortively, focally infected, and FeLV-unexposed cats is shown in Table 7.

After 20 min of incubation, the sensitivity of the POC test for the detection of anti-p15E antibodies could be increased from 31.4% to 40.0% when considering all serum samples. The sensitivity was significantly higher after 20 min of incubation compared to ten minutes (*p* < 0.001). The specificity remained the same at 96.9%. Performance parameters after 20 min of incubation time for the different countries and the different courses of infection are summarized in Table 8 and Table 9.

**Figure 2:**
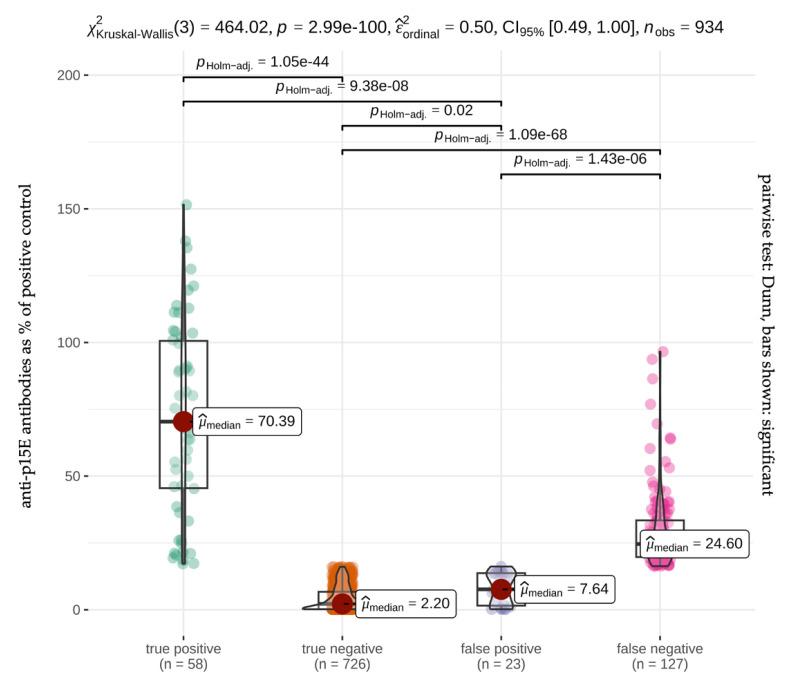
Percent deviations of median (red dot) anti-p15E antibody levels in the reference standard (enzyme-linked immunosorbent assay, ELISA). Violin plots depicting the anti-p15E antibody levels in percent for true positive, true negative, false positive, and false negative results in the point-of-care (PoC) test and the significant differences between the groups. For each box-and-whisker diagram, the solid line within the box represents the median. The lower and upper limits of the box represent the interquartile range (25th and 75th percentiles). The whiskers delimit the range; green, orange, purple, and pink dots represent the single cats. Cats with higher anti-p15E antibody levels in the ELISA (median: 70.4%) had a significantly higher chance to be detected in the PoC test than cats with lower antibody levels (median: 7.6%). In comparison, cats with low anti-p15E antibody levels in the ELISA (median: 2.2%) were significantly more often detected as a true negative in the PoC test compared to cats with higher antibody levels (median: 7.6%).

In total, 127/185 samples that tested positive in the anti-p15E ELISA were not detected in the PoC test, neither after ten minutes of incubation nor after 20 min. Particularly, samples (36/185; 19.5%) whose readings were low in the anti-p15E ELISA (close to the cut-off value; ≤20.0%) of the anti-p15E ELISA (16.3%) were not detected by the PoC test (32/36; 88.9%) (Figure 2). However, there was no significant difference in antibody concentrations in the ELISA (median: 24.6%) between false negative samples and true positive samples (median: 70.4%) in the PoC test.

## 4. Discussion

Feline leukaemia virus (FeLV) has a worldwide distribution and is one of the most important infectious agents in cats. Due to the complex pathogenesis and varying courses of FeLV infection, diagnosis is difficult and often not possible with a single test [1,2,3]. The current commercially available PoC tests for the identification of FeLV-infected cats are based on the detection of p27 antigen and thus only detect progressively infected cats. In previous studies, it has been shown that anti-p15E antibodies, in contrast to p27 antigen, are formed and can be detected in blood in most cases after exposure to FeLV regardless of the course of infection (progressive, regressive, abortive, focal) [14,18,26]. However, the reference standard (ELISA) for the detection of anti-p15E antibodies is only performed in specialized laboratories and is currently offered by only a few institutes, and ELISA results are usually only available after a few days. Therefore, a PoC test for in-house testing would be an important tool to diagnose FeLV-exposed cats immediately at the visit at the veterinarian. For potential blood donor cats, easy identification of regressively infected cats is of particular importance, as they can transmit FeLV provirus to FeLV-naive cats via blood transfusions [11]. Identification of regressively infected cats is also important in multi-cat households, as regressive infection can be reactivated to progressive infection with virus shedding, especially after suppression of the immune system. In addition, identification of FeLV-exposed cats (abortive infections) prior to vaccination might be helpful in deciding whether or not to vaccinate cats against FeLV [1,2,3]. A PoC test (v-RetroFel^®^, scil animal care company GmbH, Viernheim, Germany) for simultaneous detection of p27 antigen and anti-p15E antibody was recently evaluated in naturally FeLV-infected cats from Australia and Germany [17]. Unfortunately, the results in this previous study were not very promising concerning the detection of anti-p15E antibodies within this PoC test. Therefore, modifications were made by the manufacturer to improve the PoC test for the detection of anti-p15E antibodies.

In the present study, the modified version of the PoC test (v-RetroFel^®;^ modified version 2021) was easy and quick to perform under practical conditions due to storage at room temperature and easy and few steps required during performance. However, in approximately one third of the samples (21/58; 36.2%) that tested positive in the PoC test, the anti-p15E antibody testing resulted in only a faint colour line. A faint colour line can lead to uncertainty in result interpretation, making it challenging to determine whether the test is truly positive or negative. Faint colour lines could potentially affect the test’s accuracy. It is unlikely that the storage time and freezing/thawing of the samples could have influenced the test performance. The results of a human study showed the stability of polyclonal antibodies in serum samples during long-term storage at −65 °C and −20 °C, during multiple freeze/thaw cycles, and during shipping [31].

Regarding the detection of p27 antigen, the PoC test showed a lower sensitivity (82.8%) when considering all samples compared to the first version of the PoC test (91.3%) [17]. According to the manufacturer, no changes were made in the p27 antigen test components; nevertheless, a low sensitivity in p27 testing is a disadvantage. Especially in countries with a high prevalence of FeLV, test performance, particularly sensitivity (the ability to correctly identify serum samples as positive), is the most important parameter for preventing the spread of infection by infected cats. Furthermore, cats with false negative results do not receive the appropriate medical attention and supportive care (e.g., isolation from other cats, treatment of secondary diseases). In animal shelters and adoption programs, poor test sensitivity can lead to unrecognized adoption of FeLV-infected cats. This not only puts the newly adopted cat at risk (higher risk for secondary infections due to immunosuppression) but can also lead to the unintentional spread of the virus to other cats within the shelter or adoptive homes. Specificity for p27 antigen was high (96.0%) and thus comparable to the one of the first version of the PoC test (98.3%) [17].

Regarding anti-p15E antibodies, the sensitivity of the PoC test was increased to 31.4% compared to the first version of this test, where the sensitivity for Australian samples was 16.7% and for German samples was 8.3% [17]. All samples were interpreted both after ten minutes (according to the manufacturer’s specification) and after 20 min of incubation time to see whether an increase in sensitivity regarding anti-p15E antibodies is possible with a prolonged incubation time. With an extension of the incubation time to 20 min, the sensitivity regarding the detection of anti-p15E antibodies was increased from 31.4% to 40.0%, which is still low.

The PoC test was not able to detect most regressively, abortively, and focally infected cats that tested positive in the anti-p15E antibody ELISA (sensitivity 34.6%, 17.0%, and 20.0%). At least in samples from progressively infected cats, the PoC test showed a high sensitivity of 86.7%. However, detection of progressively infected cats through anti-p15E antibody detection is not essential as progressive infection is already detected through the presence of p27 antigen. One reason for the high sensitivity with respect to anti-p15E antibodies in progressively infected cats could be that many of these cats had high antibody concentrations (median 78.6% of positive control) in the reference standard (laboratory-based ELISA). In the present study, it was shown that cats with higher ELISA antibody concentrations (median 70.4% of positive control) were detected more reliably in the PoC test in contrast to cats with low ELISA antibody concentrations (median 24.6% of positive control). Of the 127/185 false negative samples, 116/127 samples had an ELISA antibody concentration < 50.0% of the positive control. At this point, it should be mentioned that experimentally infected cats that tested positive in the anti-p15E antibody ELISA were also all identified as positive in the PoC test. One possible reason for this could be that the experimentally infected cats all showed a quite high antibody level of over 45% of the positive control in the anti-p15E antibody ELISA. Such a high sensitivity (95.7%) and specificity (100.0%) of the p15E ELISA was already shown in a previous study [25] and was confirmed again in the present study, in which all experimentally infected cats tested positive for the presence of anti-p15E antibodies in the p15E ELISA (sensitivity: 100.0%, specificity: 100.0%). In addition, naturally infected cats could be infected with FeLV subtypes that are not detected by the PoC test. Both the laboratory-based ELISA and the PoC test used the transmembrane of FeLV subtype A (GenBank accession no. AAA93093.1) [25]. In ELISA, a cut-off of >16.3% was used as the reference standard for the detection of anti-p15E antibodies in naturally infected cats. The same applies to the PoC test, which is designed to detect samples with an anti-p15E antibody level of >16.3%. The determined cut-off values of the laboratory-based ELISA might not be appropriate for all populations or conditions, including samples from different geographic regions and different breeds or age groups of cats, factors that might have affected the applicability of the test. Westman and colleagues already suggested that the cut-off value, which determines whether a sample is considered positive, should be carefully reevaluated [17].

The specificity of anti-p15E antibody testing was 96.9% in the present study using the modified new version of the PoC test, comparable to the first version of the PoC test with a specificity of 90.2% (Australia) and 93.7% (Germany), respectively [17]. In the present study, a total of 23/934 samples were considered false positive. Contamination of the sample with substances that mimic anti-FeLV antibodies could result in false positive results. Another reason for a false-positive result in a PoC test can be the presence of antibodies against antigens that mimic the FeLV p15E epitopes and therefore cause cross-reactivity. In addition, previous vaccinations against FeLV can lead to positive results [25]. For this reason, it is important to consider the cat’s vaccination history when interpreting test results. Nevertheless, it can be concluded that the detection of anti-p15E antibodies more likely indicates a previous infection rather than vaccination. Similar results were reported in previous studies, where the majority of vaccinated cats in Switzerland had lower levels of anti-p15E antibodies than cats previously exposed to FeLV [25]. A recent study showed that the response of p15E antibodies depended on the type of FeLV vaccine administered. Cats vaccinated with an inactivated whole-virus FeLV vaccine (Fel-O-Vax^®^ Lv-K or Fel-O-Vax^®^ 5) more likely had an anti-p15E antibody response compared to those vaccinated with a subunit vaccine (Leucogen^®^) [17].

Further research is necessary to understand the clinical relevance of anti-p15E antibodies. Cats with high anti-p15E antibody levels might be protected from FeLV infection. In a previous study, it was shown that cats immunized with the FeLV transmembrane protein p15E developed neutralizing anti-p15E antibodies [32]. An experimental study attempted to include p15E in FeLV vaccines. In three of six cats immunized with p15E and experimentally infected with FeLV, protection against FeLV antigenemia was observed at day 960. The remaining three cats immunized with p15E were unprotected and developed progressive FeLV infection after experimental infection with FeLV [33].

## 5. Conclusions

The PoC test (v-RetroFel^®^; modified version 2021) provides results quickly. Especially when considering situations such as testing cats before vaccination against FeLV or screening cats for FeLV before introducing them into a new household with other felids, a quick and easy performance of the test would be essential. Sensitivity and specificity of the PoC test for FeLV anti-p15E antibody detection was improved by modifications by the manufacturer when compared to the first version of the PoC test. Nevertheless, the sensitivity was still too low. A high sensitivity and a high positive predictive value are however necessary to correctly identify cats that had contact with FeLV. Therefore, further modifications of the PoC test should be made to improve sensitivity regarding detection of anti-p15E antibodies before the test can be recommended for use under field conditions. Moreover, it would be important to further explore the clinical relevance of anti-p15E antibodies and to evaluate whether anti-p15E antibody-positive cats are protected against FeLV infection.

## Figures and Tables

**Table 1:** Classification of feline leukaemia virus infection status in 934 naturally infected cats of the present study based on the European Advisory Board on Cat Diseases FeLV diagnostic tool [29].

FeLV Infection Status	p27 Ag ELISA	Proviral DNA PCR *	Anti-p15E Ab ELISA
Progressive (n = 38)	+	+	+/−2
Regressive (n = 40)	−	+	+/−2
Abortive (n = 108)	−	−	+
Focal (n = 21)	(+) ^1^	−	+/−2
FeLV unexposed vaccinated ^∆^ (n = 72)	−	−	+/−
FeLV unexposed not vaccinated (n = 655)	−	−	−

* FeLV proviral DNA was determined in all samples using a quantitative polymerase chain reaction (qPCR), as described before [17,27]. ^∆^ Of the FeLV-unexposed cats, 72/727 (9.9%) were vaccinated against FeLV. Of the vaccinated cats, 16/72 (22.2%) tested positive in the p15E ELISA. Five of these cats were vaccinated with Purevax^®^ FeLV and two cats with Leucogen^®^; the manufacturer of the other vaccines was unknown (n = 9). ^1^ Cats with focal infection tested weakly p27 antigen-positive and proviral DNA-negative. ^2^ Not all cats with a progressive, regressive, or focal infection showed antibodies against anti-p15E. The symbol +/− means that, regardless of the course of the infection, some cats tested positive in the p15E ELISA and others tested negative in the p15E ELISA. The fields highlighted in grey indicate that the mentioned test was not used for the classification to the specific course of infection. FeLV, feline leukaemia virus; Ag, antigen; ELISA, enzyme-linked immunosorbent assay; DNA, deoxyribonucleic acid; Ab, antibody.

**Table 2:** Specificity, sensitivity, negative and positive predictive values of the point-of-care test for the detection of feline leukaemia virus p27 antigen in 60 SPF cats (30 uninfected cats, 16 progressively, and 14 regressively infected) compared to the results of an in-house developed p27 antigen enzyme-linked immunosorbent assay as a reference standard, taking experimentally infected cats into account.

p27 Antigen Positive Samples (ELISA) %	Sensitivity % (95% CI)	Specificity % (95% CI)	PPV % (95% CI)	NPV % (95% CI)
26.7	93.8 (69.8–99.8)	100.0 (92.0–100.0)	100.0 (78.2–100.0)	93.3 (88.2–99.9)

FeLV, feline leukaemia virus; ELISA, enzyme-linked immunosorbent assay; CI, confidence interval; PPV, positive predictive value (proportion of predicted positives that were true positives); NPV, negative predictive value (proportion of predicted negatives that were true positives).

**Table 3:** Specificity, sensitivity, negative and positive predictive values of the point-of-care test for the detection of feline leukaemia virus p27 antigen in comparison to the results of an in-house developed p27 antigen enzyme-linked immunosorbent assay as a reference standard, taking naturally infected cat populations from different countries into account.

Country	Prevalence of p27 Antigen (ELISA) %	Sensitivity % (95% CI)	Specificity % (95% CI)	PPV % (95% CI)	NPV % (95% CI)
All cats	6.2	82.8 (70.6–91.4)	96.0 (94.5–97.2)	57.8 (46.5–68.6)	98.8 (97.9–99.4)
Italy	10.0	92.6 (75.7–99.1)	95.0 (91.5–97.4)	67.6 (50.2–82.0)	99.1 (96.9–99.9)
Portugal	5.4	84.6 (54.6–98.1)	96.9 (93.8–98.8)	61.1 (35.8–82.7)	99.1 (96.8–99.9)
Germany	4.7	60.0 (32.3–83.7)	96.4 (93.6–98.2)	45.0 (23.1–68.5)	98.0 (95.7–99.3)
France	2.8	100.0 (29.2–100.0)	95.2 (89.1–98.4)	37.5 (8.5–75.5)	100.0 (96.3–100.0)

FeLV, feline leukaemia virus; ELISA, enzyme-linked immunosorbent assay; CI, confidence interval; PPV, positive predictive value (proportion of predicted positives that were true positives); NPV, negative predictive value (proportion of predicted negatives that were true positives).

**Table 4:** Specificity, sensitivity, negative and positive predictive value of the point-of-care test for the detection of feline leukaemia virus p27 antigen in comparison to the results of a laboratory-based p27 antigen enzyme-linked immunosorbent assay as a reference standard, taking different courses of feline leukaemia virus infection into account.

Course of FeLV-Infection	Prevalence of p27 Antigen (ELISA) %	Sensitivity % (95% CI)	Specificity % (95% CI)	PPV % (95% CI)	NPV % (95% CI)
All cats	6.2	82.8 (70.6–91.4)	96.0 (94.5–97.2)	57.8 (46.5–68.6)	98.8 (97.9–99.4)
Progressive Infection ^1^	100.0	100.0 (90.8.1–100.0)	n. d.	100.0 (90.8–100.0)	n. d.
Regressive Infection ^2^	0.0	n. d.	100.0 (91.2–100.0)	n. d.	100.0 (91.2–100.0)
Abortive Infection ^3^	0.0	n. d.	95.7 (85.5–99.5)	n. d.	100.0 (92.1–100.0)
Focal Infection ^4^	100.0	63.6 (30.8–89.1)	n. d.	100.0 (59.0–100.0)	n. d.
Unexposed	0.0	n. d.	96.1 (94.5–97.4)	n. d.	100.0 (99.5–100.0)

^1^ progressively infected cats were defined as follows: p27 antigen-positive and proviral DNA-positive. ^2^ regressively infected cats were defined as follows: p27 antigen-negative, proviral DNA-positive. ^3^ abortively infected cats were defined as follows: p27 antigen-negative, proviral DNA-negative, anti-FeLV antibody-positive. ^4^ focally infected cats were defined as follows: p27 antigen weak positive, proviral DNA-negative. FeLV, feline leukaemia virus; ELISA, enzyme-linked immunosorbent assay; CI, confidence interval; PPV, positive predictive value (proportion of predicted positives that were true positives); NPV, negative predictive value (proportion of predicted negatives that were true positives); n. d., could not be determined as none of the samples tested positive or negative in the p27 antigen enzyme-linked immunosorbent assay.

**Table 5:** Specificity, sensitivity, negative and positive predictive values of the point-of-care test for the detection of feline leukaemia virus anti-p15E antibodies in 60 SPF cats (30 uninfected, 30 FeLV-infected) in comparison to the results of a laboratory-based anti-p15E antibody enzyme-linked immunosorbent assay as a reference standard.

Anti-p15E Antibody Positive Samples (ELISA) %	Sensitivity % (95% CI)	Specificity % (95% CI)	PPV % (95% CI)	NPV % (95% CI)
50.0	100.0 (88.4–100.0)	100.0 (88.4–100.0)	100.0 (88.4–100.0)	100.0 (88.4–100.0)

FeLV, feline leukaemia virus; ELISA, enzyme-linked immunosorbent assay; CI, confidence interval; PPV, positive predictive value (proportion of predicted positives that were true positives); NPV, negative predictive value (proportion of predicted negatives that were true positives).

**Table 6:** Specificity, sensitivity, negative and positive predictive values of the point-of-care test for the detection of feline leukaemia virus anti-p15E antibodies in comparison to the results of a laboratory-based anti-p15E antibody enzyme-linked immunosorbent assay as a reference standard, taking cat populations from different countries into account.

Country	Prevalence of Anti-p15E Antibodies (ELISA) %	Sensitivity % (95% CI)	Specificity % (95% CI)	PPV % (95% CI)	NPV % (95% CI)
All cats	19.8	31.4 (24.7–38.6)	96.9 (95.4–98.0)	71.6 (60.5–81.1)	85.1 (82.5–87.4)
Italy	21.2	45.6 (32.4–59.3)	94.8 (90.0–97.4)	70.3 (53.0–84.1)	86.6 (81.6–90.7)
Portugal	22.5	27.8 (16.5–41.6)	96.7 (93.1–98.8)	71.4 (47.8–88.7)	82.2 (76.5–87.0)
Germany	14.2	20.0 (9.6–34.6)	98.2 (95.8–99.4)	64.3 (35.1–87.2)	88.2 (84.0–91.6)
France	27.1	27.6 (12.7–47.2)	98.7 (93.1–100.0)	88.9 (51.8–99.7)	78.6 (69.1–86.2)

FeLV, feline leukaemia virus; ELISA, enzyme-linked immunosorbent assay; CI, confidence interval; PPV, positive predictive value (proportion of predicted positives that were true positives); NPV, negative predictive value (proportion of predicted negatives that were true positives).

**Table 7:** Specificity, sensitivity, negative and positive predictive value of the point-of-care test for the detection of feline leukaemia virus anti-p15E antibodies in comparison to the results of a laboratory-based anti-p15E antibody enzyme-linked immunosorbent assay as a reference standard, taking different courses of feline leukaemia virus infection into account.

Course of FeLV-Infection	Prevalence of Anti-p15E Antibodies (ELISA) %	Sensitivity % (95% CI)	Specificity % (95% CI)	PPV % (95% CI)	NPV % (95% CI)
All cats	19.8	31.4 (24.7–38.6)	96.9 (95.4–98.0)	71.6 (60.5–81.1)	85.1 (82.5–87.4)
Progressive Infection ^1^	78.9	86.7 (69.3–96.2)	100.0 (63.1–100.0)	100.0 (86.8–100.0)	66.7 (34.9–90.1)
Regressive Infection ^2^	65.0	34.6 (17.2–55.7)	85.7 (57.2–98.2)	81.8 (48.2–97.7)	41.4 (23.5–61.1)
Abortive Infection ^3^	100.0	16.7 (10.2–25.1)	n. d.	100.0 (81.5–100.0)	n. d.
Focal Infection ^4^	23.8	20.0 (0.5–71.6)	100.0 (79.4–100.0)	100.0 (2.5–100.0)	80.0 (56.3–94.3)
FeLV unexposed vaccinated	22.2	25.0 (7.3–52.4)	98.2 (90.5–99.9)	79.8 (28.4–99.5)	82.3 (70.8–90.4)
FeLV unexposed not vaccinated	0.0	n. d.	97.0 (95.3–98.1)	n. d.	100.0 (99.4–100.0)

^1^ progressively infected cats were defined as follows: p27 antigen-positive and proviral DNA-positive. ^2^ regressively infected cats were defined as follows: p27 antigen-negative, proviral DNA-positive. ^3^ abortively infected cats were defined as follows: p27 antigen-negative, proviral DNA-negative, anti-FeLV antibody-positive. ^4^ focally infected cats were defined as follows: p27 antigen weak positive, proviral DNA-negative. FeLV, feline leukaemia virus; ELISA, enzyme-linked immunosorbent assay; CI, confidence interval; PPV, positive predictive value (proportion of predicted positives that were true positives); NPV, negative predictive value (proportion of predicted negatives that were true positives); n. d., could not be determined as none of the samples tested negative in the anti-p15E antibody enzyme-linked immunosorbent assay.

**Table 8:** Specificity, sensitivity, negative and positive predictive value of the point-of-care test after 20 min incubation time to detect feline leukaemia virus anti-p15E antibodies compared with the results of a laboratory-based anti-p15E antibody enzyme-linked immunosorbent assay in cats from Italy, Portugal, Germany, and France.

Country	Prevalence of Anti-p15E Antibodies (ELISA) %	Sensitivity % (95% CI)	Specificity % (95% CI)	PPV % (95% CI)	NPV % (95% CI)
All cats	19.8	40.0 (32.9–47.4)	96.9 (95.4–98.0)	76.3 (66.6–84.3)	86.7 (84.3–89.0)
Italy	21.2	50.9 (37.3–64.4)	94.8 (90.9–97.4)	72.5 (56.1–85.4)	87.8 (82.8–91.7)
Portugal	22.5	40.7 (27.6–55.0)	96.8 (93.1–98.8)	78.6 (59.1–91.7)	84.9 (79.4–89.4)
Germany	14.2	22.2 (11.2–37.1)	98.2 (95.8–99.4)	66.7 (38.4–88.2)	88.5 (84.3–91.8)
France	27.1	44.8 (26.5–64.3)	98.7 (93.1–100.0)	92.9 (66.1–99.8)	82.8 (73.6–89.8)

FeLV, feline leukaemia virus; ELISA, enzyme-linked immunosorbent assay; CI, confidence interval; PPV, positive predictive value (proportion of predicted positives that were true positives); NPV, negative predictive value (proportion of predicted negatives that were true positives).

**Table 9:** Specificity, sensitivity, negative and positive predictive value of the point-of-care test after 20 min incubation time to detect feline leukaemia virus anti-p15E antibodies compared with the results of a laboratory-based anti-p15E antibody enzyme-linked immunosorbent assay, in progressively, regressively, abortively, focally, and unexposed cats.

Course of FeLV-Infection	Prevalence Anti-p15E Antibodies (ELISA) %	Sensitivity % (95% CI)	Specificity % (95% CI)	PPV % (95% CI)	NPV % (95% CI)
All cats	19.8	40.0 (32.9–47.4)	96.9 (95.4–98.0)	76.3 (66.6–84.3)	86.7 (84.3–89.0)
Progressive infection ^1^	78.9	90.0 (73.5–97.9)	100.0 (63.1–100.0)	100.0 (87.2–100.0)	72.7 (39.0–94.0)
Regressive infection ^2^	65.0	42.3 (23.4–63.1)	85.7 (57.2–98.2)	84.6 (54.6–98.1)	44.4 (25.5–64.7)
Abortive infection ^3^	100.0	28.7 (20.4–38.2)	n. d.	100.0 (88.8–100.0)	n. d.
Focal infection ^4^	23.8	20.0 (0.5–71.6)	100.0 (79.4–100.0)	100.0 (2.5–100.0)	80.0 (56.3–94.3)
FeLV unexposed vaccinated	22.2	31.3 (11.0–58.7)	98.2 (90.5–99.9)	83.2 (35.9–99.6)	83.5 (72.1–91.4)
FeLV unexposed not vaccinated	0.0	n. d.	97.0 (95.3–98.1)	n. d.	100.0 (99.4–100.0)

^1^ progressively infected cats were defined as follows: p27 antigen-positive and proviral DNA-positive. ^2^ regressively infected cats were defined as follows: p27 antigen-negative, proviral DNA-positive. ^3^ abortively infected cats were defined as follows: p27 antigen-negative, proviral DNA-negative, anti-FeLV antibody-positive. ^4^ focally infected cats were defined as follows: p27 antigen weak positive, proviral DNA-negative. FeLV, feline leukaemia virus; ELISA, enzyme-linked immunosorbent assay; CI, confidence interval; PPV, positive predictive value (proportion of predicted positives that were true positives); NPV, negative predictive value (proportion of predicted negatives that were true positives); n. d., could not be determined as none of the samples tested negative in the anti-p15E antibody enzyme-linked immunosorbent assay.

## Data Availability

All data presented in this paper are available upon request.
